# Mother–Infant and Extra-Dyadic Interactions with a New Social Partner: Developmental Trajectories of Early Social Abilities during Play

**DOI:** 10.3389/fpsyg.2017.00436

**Published:** 2017-04-11

**Authors:** Roberta Fadda, Loredana Lucarelli

**Affiliations:** Department of Pedagogy, Psychology, Philosophy, University of CagliariCagliari, Italy

**Keywords:** mother-infant feeding and play interactions, new social partner in extra-dyadic play interactions, early social communication assessment, developmental trajectories, follow-up study, intervention programs to enhance caregiver–infant relationships

## Abstract

Mother–infant interactions during feeding and play are pivotal experiences in the development of infants’ early social abilities (Stern, 1985, 1995; Biringen, 2000). Stern indicated distinctive characteristics of mother–infant interactions, respectively, during feeding and play, suggesting to evaluate both to better describe the complexity of such early affective and social experiences (Stern, 1996). Moreover, during the first years of life, infants acquire cognitive and social skills that allow them to interact with new social partners in extra-dyadic interactions. However, the relations between mother–child interactions and infants’ social skills in extra-dyadic interactions are still unknown. We investigated longitudinally the relations between mother–child interactions during feeding and play and child’s pre-verbal communicative abilities in extra-dyadic interactions during play. 20 dyads were evaluated at T_1_ (infants aged between 9–22 months) and 6 months later, at T_2_. The interdyadic differences in mother–infant interactions during feeding and play were evaluated, respectively, with the “Feeding Scale” (Chatoor et al., 1997) and with the “Play Scale” (Chatoor, 2006) and the socio-communicative abilities of children with a new social partner during play were evaluated with the “Early Social Communication Scales” (Mundy et al., 2003). We distinguished the dyads into two categories: dyads with functional interactions (high dyadic reciprocity, low dyadic conflict) and dyads with dysfunctional interactions (lower dyadic reciprocity, higher dyadic conflict). At T_1_, infants belonging to dyads with dysfunctional interactions were significantly lower in “Initiating Joint Attention” and in “Responding to Joint Attention” in interaction with a new social partner compared to the infants belonging to dyads with functional interactions. At T_2_, infants belonging to dyads with dysfunctional interactions were significantly lower in “Initiating Social Interactions” with a new social partner compared to the infants belonging to dyads with functional interactions. There were significant correlations between the quality of mother–infant interactions during feeding and infants’ social abilities in interaction with a stranger both at T_1_ and at T_2_. This study showed a stable relation over time between mother–child interactions and child’s social communicative skills in extra-dyadic interactions.

## Introduction

This study explores the developmental trajectories of mother–infant interactions during feeding and play and of extra-dyadic interactions with a stranger during play in the first 2 years of life. The theoretical and empirical framework is the Infant Research, which combines the attachment theory with the developmental models of intersubjectivity (Stern, 1985; Trevarthen and Aitken, 2001; Lavelli, 2007; Papoušek, 2007; Ammaniti and Gallese, 2014). According to this framework, the infants’ social communication abilities emerge within the context of functional dynamic interexchanges between infants and caregivers (Dunst et al., 1990; Sameroff, 2010).

From this perspective, the quality of mother–infant interactions during the first years of life is grounded both on the infant natural predisposition to socially interact with their partners and on the mother’s behavior and her emotional availability (Stern, 1995; Biringen, 2000; Papoušek, 2007). The most important and well-known feature of maternal behavior is sensitivity to the infants cues. Mothers who accurately perceive and respond to the infant needs, distress and communication efforts are more likely to promote the infants’ socio-communicative abilities than the mothers who ignore, reject or respond inconsistently to the infant needs and communication bids (Ainsworth et al., 1978; Teti and Candelaria, 2002; Meins et al., 2011). This sensitivity might promote a mutual mother–infant engagement, whit a beneficial effect on infants’ socio-communicative development (Teti and Candelaria, 2002).

Mother’s sensitivity promotes high levels of infant cooperation with the mother behavior toward the infant during the first years of life (Ainsworth et al., 1978). At the same time, the quality of maternal behavior is bound up by infant characteristics, so that a mother is sensitive as long as she is able of modifying her behavior in response to the infant’s individual characteristics and needs. Moreover, maternal emotional availability plays a preeminent role in the development of mother-infant healthy adaptation (Emde, 1980; Biringen, 2000). The parental emotional availability view (Biringen, 2000; Emde, 2000) of sensitivity (Ainsworth et al., 1978) emphasizes several significant aspects of parental sensitivity, like the affective level of the interactions, the negotiation of the intra-dyadic conflicts and the dyssynchronous interactions, including the successful repair of such situations (Tronick and Cohn, 1989; Biringen et al., 1997). As shown by recent studies, mother–infant interactions are not characterized by continuous synchrony, but the ongoing regulation within the dyad can vary in terms of degrees of coordination, disruption and repair, and maternal flexibility to manage this variability (Beebe and Lachmann, 1994).

From the theoretical and empirical perspective described so far, mother–infant interactions during feeding and play have been intensively studied as the ideal contexts to promote children socio-communicative abilities (Stern, 1995, 1996; Biringen, 2000; Meins et al., 2011). In the course of the uncountable mutual exchanges during feeding and play activities with their mother, infants not only practice a number of social skills like turn-taking and joint attention behaviors, but they also develop self-regulatory abilities and a basic sense of self (Fogel, 1995; Fonagy et al., 2002; Feldman, 2007; Ammaniti and Gallese, 2014; Lucarelli et al., 2017).

However, reviewing the literature, we have found that little systematic attention has been given in order to determine whether the quality of mother–infant interaction is related to the infant social abilities in interaction with a stranger in extra-dyadic contexts. Only five studies to date, for the best of our knowledge, explored this relationship; all these studies used the Early Social Communication Scales (E.S.C.S.), a structured observation developed by Mundy et al. (2003) in order to measure the infant’s social communicative competencies in extra-dyadic interactions.

In a longitudinal study, Markus-Meyer et al. (2000) demonstrated that the infants’ abilities to respond to joint attention correlates with the joint attention episodes during mother–infant interaction in a free play context at 18 months.

Another study (Crowson, 2001) indicated that attachment at 15 months predicts joint attention exchange during mother–infant interactions at 24 months but only in relation to the infants’ early joint attention abilities in interaction with a stranger. Meins et al. (2011) showed that, at 15 months, initiating joint attention with an experimenter is associated with insecure-avoidant attachment. These results seem to indicate that insecure-avoidant infants may compensate for reduced social contact with the caregiver by producing more initiating joint attention behaviors during the interaction with a stranger compared to infants with secure attachment. Another study (Farhat, 2010) evidenced that responding to joint attention predicts language development at 24 months but only in infants belonging to dyads with low levels of maternal intrusiveness.

Finally, a recent study confirmed the relationship between the quality of mother–infant interaction and the infant’s social communicative competencies in extra-dyadic interactions (Fadda et al., 2014). Infants aged between 9 to 24 months were observed in interaction with the mother during feeding and play and in interaction with a stranger with the E.S.C.S. The results indicated a negative correlation between high levels of interactive conflict in mother–infant interaction during feeding and play and the ability of the infant to respond to joint attention behaviors in an extra-dyadic context.

In summary, the five studies illustrated so far indicated a relationship between the quality of mother–infant interaction and the infants’ social abilities in interaction with a stranger. However, these studies are still spare and need confirmation. Moreover, it is still unknown whether infants’ social abilities in extra-dyadic contexts are related with the quality of mother–infant interaction across two time points.

### Aims of the Study

This study aimed to expand a previous cross-sectional research data (Fadda et al., 2014), by investigating prospectively the relationship between mother–infant interactions and infant social competencies with a stranger across two time points: at T_1_, when the infants were aged between 9 and 22 months, and 6 months later, at T_2_. We chose to investigate the relationship between mother–infant interactions and infant social competencies specifically 6 months later because this is the same as that for the longitudinal assessment by means of the Feeding and Play Scales (Chatoor et al., 1998; Chatoor, 2006) used in previous studies. This interval has also been frequently used in the studies that used the Early Social Communications Scales (Mundy et al., 2007). This time interval takes into account the speed at which the developmental changes in the abilities considered are expected to occur in order to ascertain the process of development. Considering the wide age range of participants at T1, this study needs to be considered as exploratory. We assessed a sample of mothers without a current psychopathological condition in order to explore the relations between the quality of mother–infant interactions and infant social skills in absence of maternal psychopathological illness, which is a well-known risk factor for infant development (see for a review: Seifer and Dickstein, 2000).

## Materials and Methods

### Participants

The present study examined 20 dyads at T_1_ when the children (19 males) aged between 9–22 months (mean age = 14 months; *SD* = 3.873) and at T_2_ (infants aged between 15–28 months; mean age = 20 months; *SD* = 3.873). The current study continues a cross-sectional research, previously published (Fadda et al., 2014), by evaluating the dyads of the original sample. 10 of the 30 dyads of the original sample dropped-out at T2 and, therefore, they were not considered in this prospective study. Participants were recruited in two public childcares in the city of Cagliari. Mothers were aged between 30 and 43 years (mean age = 35; *SD* = 4). The gestational age and the development of all children were in the normal range, and they were all the only child of the family. All children were breast-fed and weaned at T_1._ Their mothers did not show psycho-pathological symptoms as evaluated at the beginning of the two time points by the Psychiatric Symptom Checklist-90-Revised (Derogatis, 1994). The dyads belonged to the middle/middle-high socioeconomic level, according to the Hollingshead’s social status index (1975).

### Ethics Statement

Informed written consent was obtained from the parents. The study was approved by the ethics committee of the Department of Pedagogy, Psychology, Philosophy of the University of Cagliari (Italy) and it was carried out in accordance with the Society for Research in Child Development’s (SRCD) Ethical Standards for Research with Children, the Italian Psychological Association’s Ethical Standards for Research with Humans, and the World Medical Association’s Helsinki Declaration, as revised on October 2008.

### Measures

#### Feeding Scale-Observational Scale for Mother–Infant Interaction during Feeding

Each dyad was observed for 20 min at the usual time of one of the main meals of the children. The mothers were invited to behave as they usually do with their children at home (Chatoor et al., 1997) and mother–child interaction was video-recorded and coded according to the Feeding Scale-Observational Scale for Mother–Infant Interaction during Feeding (Chatoor et al., 1997), in the Italian version “Scala di Valutazione dell’Interazione Alimentare Madre-Bambino – S.V.I.A.” (Lucarelli et al., 2002; Ammaniti et al., 2006, unpublished). The S.V.I.A. includes 41 items, representing four subscales: Affective State of the Mother, Interactional Conflict, Food Refusal Behavior of the Child, and Affective State of the Dyad. Each item received a score on a Likert scale of 0 (*none*), 1 (*a little*), 2 (*pretty much*), and 3 (*very much*); a global rating is obtained for each subscale.

##### Affective state of the mother subscale (15 items)

Affective State of the Mother subscale (15 items) refers to both the possible difficulties of the caregiver in showing positive affect and the frequency and quality of negative affect. It also evaluates the mother’s ability to interpret the child’s signals and facilitate reciprocal and empathic exchanges. The higher the rate in this subscale, the greater the number of the mother’s difficulties in expressing positive feelings and in correctly interpreting and tuning according to the infants’ needs. Some examples of the items of this subscale are: “Mother shows pleasure toward infant in gaze, voice, or smile”; “Mother positions infant for reciprocal exchange”; “Mother appears cheerful”; “Infant smiles at mother”; “Mother appears sad”; “Infant avoids gaze.”

##### Interactional conflict subscale (16 items)

Interactional Conflict subscale (16 items) evaluates both the presence and intensity of exchanges of conflict within the dyad. The overall number of points is high when, for example, the mother forces the child to eat, she is not flexible in regulating pauses and turn-taking with the child, and she directs the meal according to her own emotions and intentions rather than following the communicative feedback of the child whereas the child shows behaviors of distress and avoidance of feeding exchanges in response to the intrusiveness of the mother. Some examples of the items of this subscale are: “Mother controls feeding by overriding infant’s cues”; “Mother misses infant’s cues”; “Mother interrupts or terminates feeding causing distress in infant”; “Infant refuses to open the mouth”; “Infant cries when food offered”; “Mother appears distressed”; “Infant appears distressed.”

##### Food refusal behaviors of the child subscale (4 items)

Food Refusal Behaviors of the Child subscale (4 items) explores the feeding patterns of the child, indicating food refusal, poor nutritional intake, and difficult regulation of state such as irritability and/or hyperexcitability, being easily distracted, showing opposition, and negativity. This subscale also examines non-contingent maternal behaviors during feeding ex-changes (i.e., when the mother is not able to share the child’s rhythms and arbitrarily interrupts the meal, causing discomfort to the child). A high rate indicates a lack of reciprocal adaptation between the two partners and a high frequency of child’s food refusal behaviors. Some examples of the items of this subscale are: “Infant turns away from food”; “Infant arches from food”; “Infant appears easily distracted during feeding.”

##### Affective state of the dyad subscale (6 items)

Affective State of the Dyad subscale (6 items) evaluates the quality of affect in the mother–child interaction. A high rate indicates a negative involvement in the dyad, in which emotions of anger and hostility prevail. In this situation, the caregiver does not facilitate the child’s autonomous initiatives by exerting a constant control. The child is intensely reactive, showing distress. Some examples of the items of this subscale are: “Mother waits for infant to initiate interactions”; Mother forces bottle or food into infant’s mouth”; “Mother distracts or allow infant to distract during feeding”; “Child appears angry.”

The Feeding Scale (Chatoor et al., 1997, 1998) and the Italian version S.V.I.A. evaluate the quality of mother–infant interactions to highlight infants and toddlers that need to be seen for further clinical evaluations. However, this scale also indicates a borderline cut-off, which indicates transient dysfunctional interactions that “should be watched over time,” even though the dysfunctions are not of concern at the moment.

The borderline cut-off allows to distinguish the dyads into two categories:

##### Dyads with functional interactions

*T*-scores lower than 60 in each of the four subscales indicate an interaction characterized by reciprocity, positive affect, low conflict and infant’s self-regulatory abilities during feeding.

##### Dyads with dysfunctional interactions

*T*-scores between 60 and 70 in two of the four subscales of the Feeding Scale indicate a condition of transient dysfunctional interaction that “should be watched over time.”

Studies carried out for psychometric properties have confirmed satisfactory inter-rater reliability, construct, and discriminant validity for this tool (Chatoor et al., 1997; Lucarelli et al., 2002; Ammaniti et al., 2004a,b, 2010). In the Italian version, the inter-rater reliability, estimated with the use of intraclass correlation coefficients, was from Pearson’s *r* = 0.82, *p* ≤ 0.01, to Pearson’s *r* = 0.92, *p* ≤ 0.01. The discriminant analysis used to assess the ability of the Feeding Scale to predict group membership of normally developing children (vs. children with feeding disorders) showed correct group classification ranging from 82 to 92% (Ammaniti et al., 2004a,b, 2010).

#### Parent-Child Play Scale

After the meal, mother–infant interaction was observed during free play. During 10-min play session, mothers were provided a standardized set of age appropriate toys and were instructed to play with their children as they would at home, according to the procedure indicated by Chatoor (2006). Mothers and children could play with the following toys: a doll, a baby bottle, colored blocks, a book of figures and a shape sorter toy. The mother–child interaction in the Play Scale includes 32 items representing four subscales: Dyadic Reciprocity, Maternal Unresponsiveness to the Infant’s/Toddler’s Cues, Dyadic Conflict and Maternal Intrusiveness (Chatoor, 2006); the Italian version “Scala di Valutazione dell’Interazione di Gioco Madre-Bambino” was used (Lucarelli and Cimino, 2008). Each item received a score on a Likert scale of 0 (*none*), 1 (*a little*), 2 (*pretty much*), and 3 (*very much*). If the behavior did not occur, it was rated as 0 (none); if the behavior was observed sometimes or rarely, it was rated as 1 (a little); if the behavior occurred several times, it was rated as 2 (pretty much); and if the behavior occurred often or repeatedly throughout the observational period, it was rated as 3 (pretty much).

##### Dyadic reciprocity subscale (15 items)

Dyadic Reciprocity subscale (15 items) evaluates the quality of the mother–infant interaction in terms of positive affect and synchrony and reflects the quality of relatedness and affective engagement between the mother and child. Both the mother and infant spontaneous bids to initiate social interactions are considered. The rating system of this scale was reversed, with higher scores indicating low dyadic reciprocity. Some examples of the items of this subscale are: “Parent attends to the infant’s play”; “Parent enjoys interacting with the infant”; “Parent makes encouraging remarks about the infant’s play”; “Child looks at parent”; “Infant plays with parent.”

##### Maternal unresponsiveness to infant’s/toddler’s cues subscale (6 items)

Maternal Unresponsiveness to Infant’s/Toddler’s Cues subscale (6 items) refers to the degree of which a parent fails to be contingent and to support the infant’s play activities and appears unaware of the child’s ongoing activities during play. Some examples of the items of this subscale are: “Parent positions or holds infant with restriction of normal movement”; “Parent is unaware of the infant’s activities”; “Parent appears detached and/or withdrawn from the infant.”

##### Dyadic conflict subscale (6 items)

Dyadic Conflict subscale (6 items) refers to the degree to which the parent displays anxiety, distress, anger, and/or makes negative or critical remarks about the child or criticizes the child’s play, and the degree to which the child appears distressed, and/or angry during the entire observational period; this subscale evaluates the mother-infant conflict during play and the difficult in cooperating during the interaction. Some examples of the items of this subscale are: “Parent appears distressed”; “Parent appears angry”; “Parent makes negative or critical remarks about the infant’s play”; “Infant appears distressed.”

##### Maternal intrusiveness subscale (5 items)

Maternal Intrusiveness subscale (5 items) describes the extent to which the mother handles her child unnecessarily, acts arbitrarily and is disruptive to the child’s ongoing activities, or the extent to which the parent directs the child’s play verbally and/or physically, or the parent’s behaviors are not consistent with the child’s interests or cues; this subscale evaluates the maternal intrusiveness and the mother’s difficult in supporting the infant’s spontaneous cues to initiate social interaction and her/his attempts to be autonomous during play. Some examples of the items of this subscale are: “Parent directs infant to do or not to do”; “Parent controls infant’s play without regard for infant’s cues”; “Parent waits for infant to initiate interactions.”

#### Early Social Communication Scales (E.S.C.S.)

The Early Social Communication Scales – E.S.C.S. (Mundy et al., 2003) is a structured observation, which lasts 20 min, aimed to assess the individual differences in the infant’s preverbal socio-communication abilities between 8 and 30 months. The infant and the unfamiliar adult seat to the opposite sides of a tall table. Four posters (60 cm × 90 cm) were positioned, respectively, on the right, back right, on the left, back left. The experimenter presented to the child a series of 17 activities, aimed to elicit the infants’ socio-communicative behaviors, both in initiating and in responding to the adult’s communicative behaviors. The E.S.C.S. evaluates infants’ early social abilities from an interactional perspective, in which joint attention abilities not only predicts infants’ language development but also their abilities to represent the content of others’ mind (Mundy, 1995). Previous studies indicated a high inter-rater reliability of the E.S.C.S., ranging from 0.83 to 0.97 (Mundy et al., 1995, 2003). An example of the activity is the Object Spectacle Tasks, in which three wind-up mechanical toys and three hand-held mechanical toys (balloon, squeeze toy, cone toy, bellows toy are presented. In each presentation, the tester activates the toy on the table in front of, but out of reach of the child. Toys should be wound up enough to remain active for at least 6 s but not so long that the child loses interest.

The child’s social-communicative abilities were coded into three subscales:

##### Joint attention behaviors

The infant shares a common focus of attention with the adult by establishing eye contact, showing an object, giving and object or by following the direction of the adult pointing.

##### Behavioral requests

The infant requests an object or an event. For example, the infant might point toward a target object and/or give an object to the experimenter.

##### Social interactions

The infant engages in turn-taking activities and in reciprocal social interactions, like for example tickle or singing.

Furthermore, the behavior of the child in each subscale was distinguished into two subcategories: ⟨initiating⟩ a social interaction or ⟨responding⟩ to the experimenter communicative behavior.

### Procedure

The dyads were evaluated at T1 when the children (19 Boys) aged between 9–22 months (mean age = 14 months; *SD* = 3), and after 6 months at T2. Each dyad has been observed at home during feeding and free play. Mother–child interactions during feeding and playing were examined using the “Feeding Scale” (Chatoor et al., 1998; Lucarelli et al., 2002; Ammaniti et al., 2006, unpublished) and the “Play Scale” (Chatoor et al., 1997; Chatoor, 2006; Lucarelli and Cimino, 2008). The dyads were observed at the time in which the child usually ate (between 11:00 and 12:00 am). Moreover, we analyzed the socio-communicative abilities of the toddlers with a new social partner using the “Early Social Communication Scales” (Mundy et al., 2003). The E.S.C.S. were administered 2–3 days after the other two observations at the Laboratory of the Department of Pedagogy, Psychology, Philosophy of the University of Cagliari, at the same time interval of the observation of the feeding and of the play (between 11:00 and 12:00 am). The observational data were coded by independent coders, trained and certified in the use of the Feeding and Play Scales, and the E.S.C.S.

### Inter-rater Reliability

Encodings were performed by two independent observers for each of the assessment tools used. At T1, the percentage of agreement between observers, calculated on 25% of the videotaped material, was the 79% for the SVIA, 89% for the Play Scale and 80% for the ESCS. At T2, the percentage of agreement between observers, calculated on 25% of the videotaped material, was the 80% for the SVIA, 88% for the Play Scale and 82% for the ESCS.

## Results

The results indicated that the majority of the dyads showed functional interactions at T1 (11 dyads) and at T2 (12 dyads). As shown in **Table 1**, some dyads changed the nature of their interaction: 3 of the dyads that showed functional interactions at T1 showed dysfunctional interactions at T2, while 4 of the dyads that showed dysfunctional interactions at T1 showed functional interactions at T2. These results, even so preliminary considering the low number of participants included in this study, seem to indicate the transient nature of the mother–infant interaction during feeding at this early age, characterized by the challenges of the developmental pathways through a process that requires to reach an equilibrium between attachment to the caregiver and emerging autonomy, according to age and developmental stage.

**Table 1:** Frequency of dyads with functional and with dysfunctional interactions at T1 and T2.

		Feeding Scale T_2_		
		
		Functional interactions	Dysfunctional interactions	Total
Feeding Scale T_1_	Functional interaction	8	3	11
	Dysfunctional interaction	4	5	9
Total		12	8	20


**FIGURE 1:**
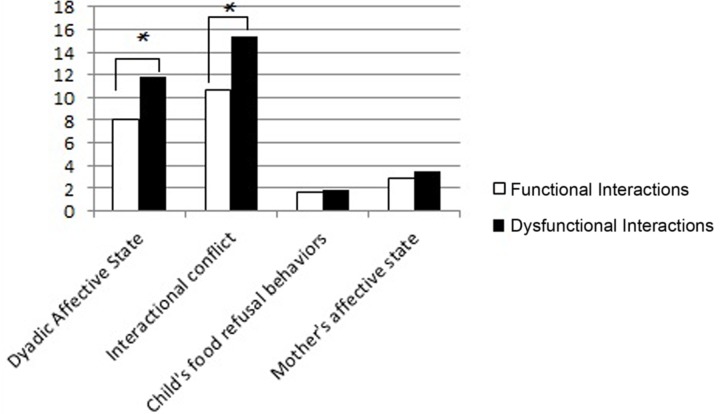
**Mean scores of dyads with functional and dysfunctional interactions at T1 in the four subscales of the Feeding Scale.** The *p*-value is reported for the comparison between the dyads with functional and dysfunctional interactions (Mann–Whitney test for independent samples; ^∗^*p* < 0.05).

At T1, the dyads with dysfunctional interactions were characterized by higher level of negative affect (Mann–Whitney *U*-test = 11.50, *p* = 0.002) and higher level of interactional conflict (Mann–Whitney *U*-test = 7.50, *p* = 0.001), compared to the dyads with functional interactions in the Feeding Scale (**Figure 1**).

**FIGURE 2:**
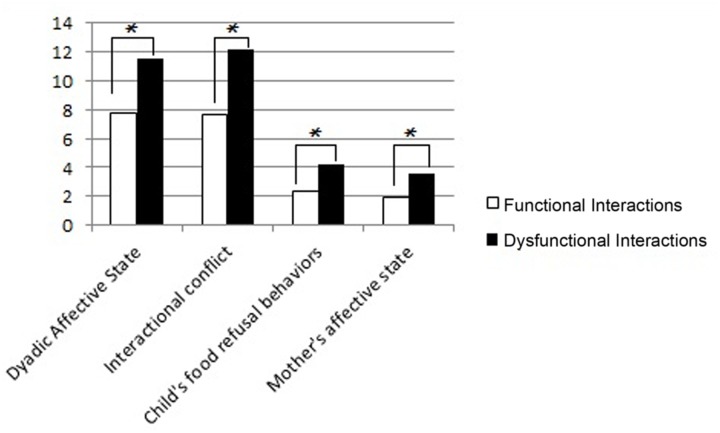
**Mean scores of dyads with functional and dysfunctional interactions at T2 in the four subscales of the Feeding Scale.** The *p*-value is reported for the comparison between the dyads with functional and dysfunctional interactions (Mann–Whitney test for independent samples; ^∗^*p* < 0.05).

At T2 (**Figure 2**), the dyads with dysfunctional interactions between mother and his/her child showed higher scores in all the subscales of the Feeding Scale compared to the dyads with functional interactions: higher levels of negative affect (Mann–Whitney *U*-test = 7.50, *p* = 0.001), interactional conflict (Mann–Whitney *U*-test = 7, *p* = 0.001), food refusal behaviors (Mann–Whitney *U*-test = 9.50, *p* = 0.002) and maternal distress (Mann–Whitney *U*-test = 13.50, *p* = 0.005). Both the results at T1 and T2 indicate interdyadic differences during feeding, with the dyads with dysfunctional interaction showing more aversive interactional conditions even in absence of maternal psycho-pathological symptoms.

**FIGURE 3:**
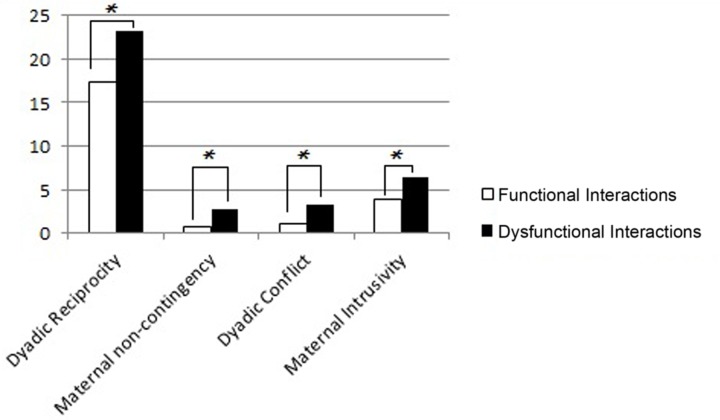
**Mean scores of dyads with functional and dysfunctional interactions at T1 in the four subscales of the Play Scale.** The *p*-value is reported for the comparison between the dyads with functional and dysfunctional interactions (Mann–Whitney test for independent samples; ^∗^*p* < 0.05).

The analysis of the mother–infant interaction during play at T1 (**Figure 3**) of the dyads with dysfunctional interactions showed lower dyadic reciprocity (Mann–Whitney *U*-test = 19.50, *p* = 0.020), higher maternal non-contingency (Mann–Whitney *U*-test = 11, *p* = 0.002), higher dyadic conflict (Mann–Whitney *U*-test = 12, *p* = 0.003) and higher maternal intrusivity (Mann–Whitney *U*-test = 10.50, *p* = 0.002), compared to the ones with functional interactions.

**FIGURE 4:**
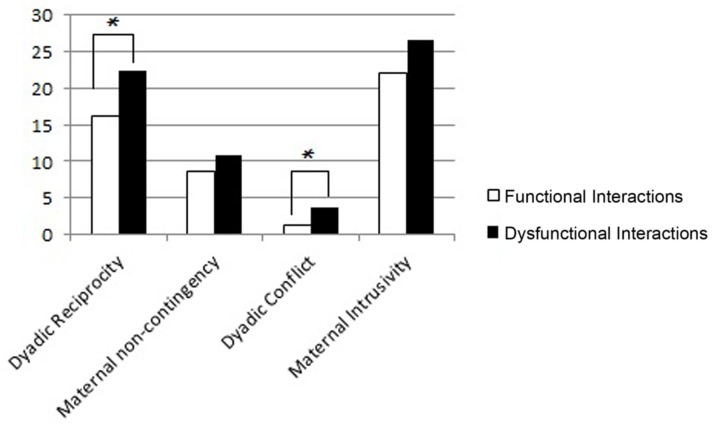
**Mean scores of dyads with functional and dysfunctional interactions at T2 in the four subscales of the Play Scale.** The *p*-value is reported for the comparison between the dyads with functional and dysfunctional interactions (Mann–Whitney test for independent samples; ^∗^*p* < 0.05).

At T2 (**Figure 4**), the dyads with dysfunctional interactions showed lower dyadic reciprocity (Mann–Whitney *U*-test = 13,50, *p* = 0.005) and higher dyadic conflict (Mann–Whitney *U*-test = 10, *p* = 0.002), compared to the mothers with functional interactions. These results, both at T1 and T2, confirm also in play more aversive interactional conditions in the dyads with dysfunctional interaction compared to the dyads with functional conditions.

**FIGURE 5:**
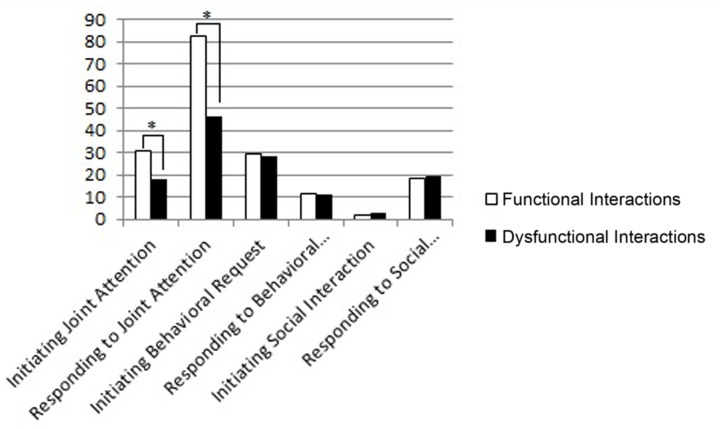
**Mean scores of dyads with functional and dysfunctional interactions at T1 in the subscales of the ESCS.** The *p*-value is reported for the comparison between the dyads with functional and dysfunctional interactions (Mann–Whitney test for independent samples; ^∗^*p* < 0.05).

We also evaluated the infants’ socio-communicative abilities with a stranger using the E.S.C.S. in the dyads with functional interactions vs. the dyads with dysfunctional interactions at T1 and T2. The results indicated that, at T1, the infants in the dyads with functional interactions showed higher scores in Initiating Joint Attention (Mann–Whitney *U*-test = 20, *p* = 0.043) and in Responding to Joint Attention (Mann–Whitney *U*-test = 14, *p* = 0.010), compared to the infants in the dyads with dysfunctional interactions (**Figure 5**).

**FIGURE 6:**
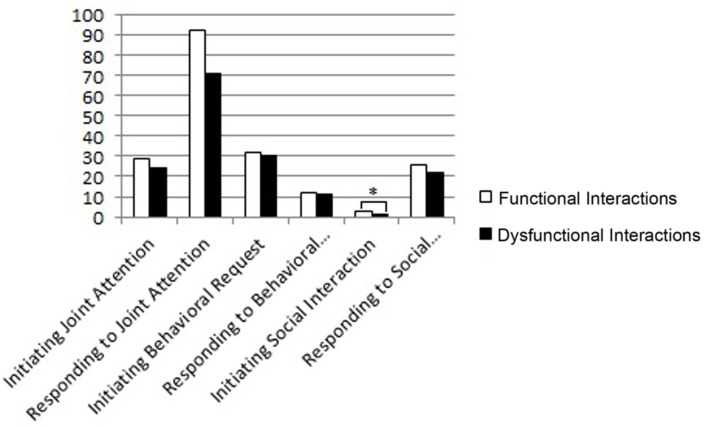
**Mean scores of dyads with functional and dysfunctional interactions at T2 in the subscales of the ESCS.** The *p*-value is reported for the comparison between the dyads with functional and dysfunctional interactions (Mann–Whitney test for independent samples; ^∗^*p* < 0.05).

At T2 (**Figure 6**), the infants in the dyads with functional interactions showed higher scores in Initiating Social Interaction (Mann–Whitney *U*-test = 23, *p* = 0.05), a class of social behaviors including very sophisticated social abilities, like for example offering a toy to the adult and/or reciprocity in the playful use of the objects. The results at T1 and T2 indicate higher social abilities in the infants’ belonging to the dyads with functional interaction.

To better explore the relationship between the quality of mother–infant interaction and the infants’ social communicative abilities in the entire sample at T1 and T2, we correlate the infants’ scores in the E.S.C.S. with the dyadic scores in the Feeding Scale and in the Play Scale. The results indicated negative significant correlations between the maternal affective state and the infants’ ability to respond to joint attention (*r* = -0.557; *p* < 0.05) and between the interactional conflict and the infants’ ability to both initiating (*r* = -0.466; *p* < 0.05) and responding (*r* = -0.510; *p* < 0.05) to joint attention at T_1_. We found a negative correlation between the maternal affective state (the higher the score, the higher the levels of anger and hostility) and the infants’ ability to respond to social interaction (*r* = -0.517; *p* < 0.05). There were no significant correlations between mother-infant behaviors during play and the infants’ social communicative abilities in the E.S.C.S. at T_1_.

At T2, the interactional conflict between mother and infant during feeding was negatively correlated with the infants’ ability to initiating social interaction (*r* = -0.500; *p* < 0.05). There was also a negative correlation between dyadic reciprocity (the higher the scores, the lower the dyadic reciprocity) during play and both the infants’ ability to initiate (*r* = -0.498; *p* < 0.05) and to respond (*r* = -0.541; *p* < 0.05) to joint attention. The maternal non-contingency during play correlated negatively with the infants’ ability to respond to joint attention (*r* = -0.453; *p* < 0.05). These correlations, both at T1 and T2, seem to indicate that functional mother–infant interactions seem to promote and support higher socio-communication abilities in the infants.

## Discussion

This study explored the developmental trajectories of mother–infant interaction and extra-dyadic interaction with a stranger in the first 2 years of life.

The results indicated that both at T1 and T2 the dyads with dysfunctional interaction showed higher level of negative affect and higher level of interactional conflict compared to the dyads with functional interaction. As the infants grew older, the dyads with dysfunctional interaction showed also more frequent infants’ food refusal behavior and higher maternal distress. These results might be explained considering the growing infants social abilities, which might foster the child’s food refusal behaviors with consequent increasing in the maternal distress. An alternative explanation might be related with the nature of the food consumed by the infants and the developmental feeding patterns. In fact, getting older, the infants are exposed to new tastes and more demanding tasks during feeding, like the use of the tools to eat. Unfortunately, we did not collected information about the infants’ eating habits. This is certainly a significant mediating factor which needs to be included in further study.

When we considered mother–infant interaction during play, we found lower levels of sensitivity in the mothers of the dyads with dysfunctional interaction compared to the ones with functional interaction at T1. At T2, the dyads with dysfunctional interaction were as appropriate as the dyads with functional interaction in contingency and intrusivity. These results might be explained considering that the differences between the context of feeding and the context of play. We can speculate that, in the feeding contexts, sometimes the food might present some aversive characteristics for the child, like for example the consistency and/or the smell, which might elicit child refusals behaviors. Moreover, in this context, the need to feed the child might push the mother to behave in a more directive way, ignoring the protests of the child. On the contrary, during play the toys are usually pleasant and desired objects for the infants, and therefore they might be easily accepted by the child. Moreover, playing together is a self-fulfill activity, so that the mother doesn’t feel any need to direct or redirect the child to achieve any particular goal rather than just having fun together. However, these interpretations are mere speculative, since we don’t know anything about the eating routines of the children. In addition, we did not evaluated whether during play the mother felt or not any need to direct or redirect the child. Thus, new studies are necessary to confirm these explanations.

In line with previous studies (Stern, 1995, 1996), our study indicates a coherence in the quality of mother–infant interaction during feeding and play. The mothers of the dyads with dysfunctional interaction were insensitive to the infants’ needs and communicative bids and showed negative affect in the course of the interaction in both contexts.

Our results also indicated a relationship between early socio-communicative abilities and the quality of mother–infant interaction over time. The infants in the dyads with functional interaction showed higher scores in Initiating Joint Attention and in Responding to Joint Attention compared to the infants in the dyads with dysfunctional interaction at T_1_. At T_2_, the infants in the dyads with functional interaction showed higher scores in Initiating Social Interaction, a class of social behaviors including very sophisticated social abilities, like for example offering a toy to the adult and/or reciprocity in the playful use of the objects. Moreover, we found significant negative correlations between the interactional conflict during feeding and the infants’ socio-communicative abilities.

Our study also confirmed that intrusiveness and interdyadic conflict were negatively correlated with the infants’ social abilities in interaction with an adult in an extra-dyadic context, as indicated by the few studies that investigated this relationship (Markus-Meyer et al., 2000; Crowson, 2001; Farhat, 2010; Fadda et al., 2014). We also found that this relationship is stable over time. Taken together, these results seem to indicate that the infants involved in mother–infant interaction characterized by more aversive interactional dynamics, like higher conflict and/or lower maternal sensitivity, might be at risk to develop pivotal social communicative abilities during infancy, in comparison with more adequate developmental patterns that were observed in infants belonging to functional dyads.

Overall, the preliminary results of our longitudinal study seem promising in further encouraging future research in order to reach a better understanding of the possible links between mother–child interactions and infants’ social skills in extra-dyadic interactions.

Although it represents a novelty in the field of early mother–infant interactions explored, our current research shows also a series of limitations which could offer useful suggestions to implement future research. First, the number of children is quite limited. This hamper the possibility to generalize our results to a normal population. It might be of interest, in a future study, to investigate the same phenomenon in a large sample of participants and to investigate the same phenomenon with parametric statistical tests.

Moreover, the age range of participants at T_1_ and T_2_ is quite wide. This might have determined the influence in our results of an uncontrolled age effect, which needs to be specifically addressed in a future study. Moreover, this study rises a general issue on continuity vs. discontinuity. Even though we considered two separate moments in time, there was some overlapping in the age of the participants at T1 and T2. As a consequence, continuity in age might have exert a confounding effect in the differences between the two separate moments of time. For this reason, our current results need to be considered as exploratory and need further investigations. A future study should consider longitudinally infants more homogenous for age. Finally, other studies indicated the importance of the maternal mental model of the attachment relationship on the quality of mother–infant interaction (Ainsworth et al., 1971, 1978). Thus, attachment might be a significant mediating factor which needs to be considered in a future study.

## Conclusion

We want to focus on the implications that this field of research might have to develop new programs of intervention, aimed to enhance and to strengthen caregiver–infant relationships, supporting the caregivers to cope with the developmental pathways and “touchpoints” (Brazelton and Greenspan, 2000; Sameroff, 2010) of a young child and to prevent dysfunctional mother–infant interactions at early age. In line with the latest knowledge in this area (Berlin et al., 2005; Powell et al., 2014), our results seem to suggest that specific interventions, grounded on the framework of the attachment theory and on the empirical findings of the Infant Research, might enhance the quality of the early child–caregiver relationships and support early child development. These interventions might be a new promising way to help the caregivers to create a healthy environment for their children’s social, emotional, physical, and cognitive development and, ultimately, their autonomy as adults (Powell et al., 2014).

## Author Contributions

RF and LL contributed to the conception and the design of this study. RF and LL organized the recruitment of the sample. RF collected, analyzed, interpreted and discussed the data, with particular attention to the results related to the “Early Social Communication Scales.” RF wrote all the sections of the manuscript and she prepared the figures. LL reviewed and integrated all the sections of the manuscript. LL contributed to the interpretation of the results and to the discussion, with particular attention to the results related to the “Feeding Scale” and with the “Play Scale.” All the authors reviewed the final version of the manuscript and approved it for publication.

## Conflict of Interest Statement

The authors declare that the research was conducted in the absence of any commercial or financial relationships that could be construed as a potential conflict of interest. The reviewer AL and the handling Editor declared their shared affiliation, and the handling Editor states that the process nevertheless met the standards of a fair and objective review.
